# Integrated virtual simulation and face-to-face simulation for clinical judgment training among undergraduate nursing students: a mixed-methods study

**DOI:** 10.1186/s12909-023-04988-6

**Published:** 2024-01-05

**Authors:** Jian Yang, Wen Jie Zhou, Si Chen Zhou, Dan Luo, Qian Liu, Ai-Ling Wang, Si-Hong Yu, Xiao-Ping Zhu, Xue Yu He, Fen Hu, Bing Xiang Yang, Jie Chen

**Affiliations:** 1 Department of Thoracic Surgery, Zhongnan Hospital of Wuhan University, No. 169 Donghu Road, Wuchang District, Wuhan, 430071 Hubei China; 2 Department of Cardiology, Renmin Hospital of Wuhan University, No. 238 Jiefang Road, Wuchang District, Wuhan, 430060 Hubei China; 3 School of Nursing, Wuhan University, No. 115 Donghu Road, Wuchang District, Wuhan, 430071 Hubei China; 4 Hospital Quality and Safety Management Office, Zhongnan Hospital of Wuhan University, No. 169 Donghu Road, Wuchang District, Wuhan, 430071 Hubei China; 5 Department of Critical Care Medicine, Zhongnan Hospital of Wuhan University, No. 169 Donghu Road, Wuchang District, Wuhan, 430071 Hubei China; 6 Clinical Research Center of Hubei Critical Care Medicine, No. 169 Donghu Road, Wuchang District, Wuhan, 430071 China; 7 Center for Critical Care and Anesthesia Nursing Research, Wuhan University School of Nursing, No. 115 Donghu Road, Wuchang District, Wuhan, 430071 Hubei China; 8 Florida State University College of Nursing, 98 Varsity Way, Tallahassee, FL 32306 USA

**Keywords:** Clinical judgment, Focus groups, Patient simulation, Quasi-experimental study, Nursing education, Virtual reality

## Abstract

**Background:**

Virtual simulation and face-to-face simulation are effective for clinical judgment training. Rare studies have tried to improve clinical judgment ability by applying virtual simulation and face-to-face simulation together. This study aimed to evaluate the effect of an integrated non-immersive virtual simulation and high-fidelity face-to-face simulation program on enhancing nursing students’ clinical judgment ability and understanding of nursing students’ experiences of the combined simulation.

**Methods:**

A sequential exploratory mixed-methods study was conducted in a nursing simulation center of a university in Central China. Third-year nursing students (*n* = 122) taking clinical training in ICUs were subsequentially assigned to the integrated non-immersive virtual simulation and high-fidelity face-to-face simulation program arm (*n* = 61) or the face-to-face simulation-only arm (*n* = 61) according to the order in which they entered in ICU training. Clinical judgment ability was measured by the Lasater Clinical Judgment Rubric (LCJR). Focus group interviews were conducted to gather qualitative data.

**Results:**

Students in both arms demonstrated significant improvement in clinical judgment ability scores after simulation, and students in the integrated arm reported more improvement than students in the face-to-face simulation-only arm. The qualitative quotes provided a context for the quantitative improvement measured by the LJCR in the integrated arm. Most of the quantitative findings were confirmed by qualitative findings, including the domains and items in the LJCR. The findings verified and favored the effect of the combination of non-immersive virtual simulation and high-fidelity face-to-face simulation integrated program on enhancing nursing students’ clinical judgment ability.

**Conclusions:**

The integrated virtual simulation and face-to-face simulation program was feasible and enhanced nursing students’ self-reported clinical judgment ability. This integrated non-immersive virtual simulation and high-fidelity face-to-face simulation program may benefit nursing students and newly graduated nurses in the ICU more than face-to-face simulation only.

**Supplementary Information:**

The online version contains supplementary material available at 10.1186/s12909-023-04988-6.

## Background

Clinical judgment ability refers to applying knowledge and professional skills to a clinical situation to solve problems and is a core competency that nursing students should possess before transited to registered nurses [1, 2]. With the high acuity of patients’ conditions and various public health emergencies, the complicated care environment requires nurses to employ good judgment ability to maintain patients’ safety and improve patients’ outcomes, which requires rapid assessment and judgment of patients so that patients receive timely and effective care [3, 4]. Nursing students take advantage of clinical training in various clinical settings, including intensive care units (ICUs), to enhance their clinical judgment ability [5]. Massive lectures and limited clinical placement opportunities impede nursing students' immersion in adequate clinical experiences [6], thus hindering the training of clinical judgment ability among nursing students.

With advances in technology, new approaches to nursing education are being used in nursing curricula, such as simulation and virtual simulation, to improve the clinical judgment skills of nursing students [7, 8]. Several benefits of high-fidelity simulation have already been established, including enhanced performance during simulated resuscitation and improved triage skills [9, 10], enhanced nursing students' communication, teamwork, and ability to manage complex situations [11, 12]. A virtual simulation is an innovative approach to providing virtual clinical experiences through a digital platform where learners can complete specific tasks in various potential environments, use the information to provide assessment and care, make clinical decisions, and observe the outcomes of actions [13, 14]. Virtual simulation allows nursing students to familiarize themselves first with problems encountered in simulation by providing flexible and repetitive exercises [15]. Although previous studies have the effect of simulation-based intensive care nursing training on enhancing nursing students’ performance [9, 12], and the impact of virtual simulation training on improving clinical judgment ability in newly graduated nurses [16], rare studies have tried to improve clinical judgment ability by integrating virtual simulation and face-to-face simulation together among nursing students during the clinical training in ICUs.

The present study aimed to verify the effect of a non-immersive virtual simulation and high-fidelity face-to-face simulation integrated program and compared the effect between the intergrade program (Integrated) and the face-to-face stimulation only (Simulation) on enhancing the clinical judgment ability of third-year nursing students during clinical training in ICUs. One of the vSim for Nursing scenarios (Version 1, Wolters Kluwer, Philadelphia, PA, USA) was used in the virtual simulation (acute pulmonary embolism); this scenario (acute pulmonary embolism) was also adopted into high-fidelity face-to-face simulation scenario in the current study following the standards of best practice for simulation [17]. The hypotheses included: a) both Integrated program and Simulation could improve students’ clinical judgment ability, b) students in the Integrated arm improve more than students in the Simulation arm, and 3) the simulation was acceptable among nursing students. This study also explored students’ experiences of the integrated program to provide context for the effects of virtual simulation.

## Methods

### Study design

**Fig. 1:**
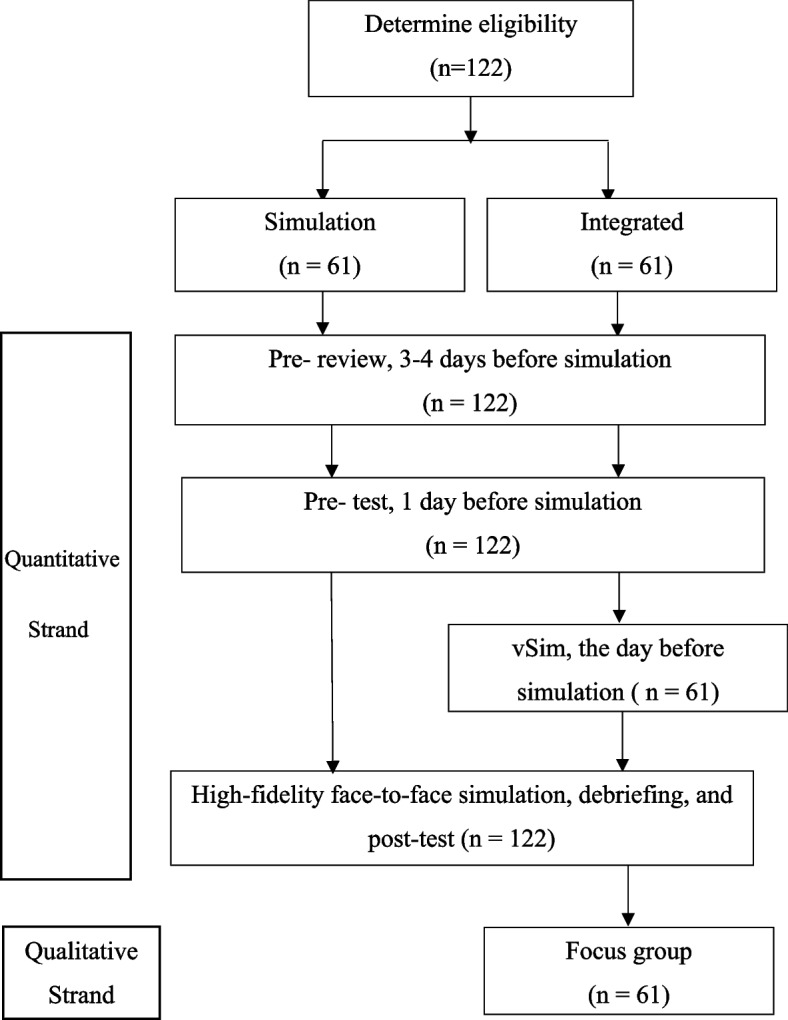
Flow chart of the study. Note: Integrated, the non-immersive virtual simulation and high-fidelity face-to-face simulation integrated program arm

An explanatory sequential mixed methods design (QUAN → qual) [18] was employed in the present study. This design consisted of quantitative analysis and qualitative description to enrich and provide context for the quantitative findings. The guideline for reported mixed-methods studies [19] was followed (Supplementary file 1). A two-group, non-randomized, pre-and post-test quasi-experiment design was conducted in the quantitative strand (Fig. 1). Focus group interviews were conducted sequentially in the qualitative strand. Inform consent was signed by each of the participants following ethics approval.

### Quantitative phase

### Setting

This study was conducted in a simulation center in central China. The Bachelor of Science in Nursing program is a four-year synchronous lecture and clinical training program. Simulation has been embedded in most nursing curricula, including health assessment, fundamentals of nursing, and clinical nursing (i.e., medical-surgical nursing, pediatric nursing, intensive care nursing). Undergraduate nursing students take some lectures and take turns completing assigned clinical training in each academic semester. The four-week clinical training in ICU was appointed in the third year, and students take turns to complete the ICU training in Fall and Spring semesters.

### The clinical training in ICU

The clinical training in ICU in the third year consisted of lectures, simulations, and clinical placement in each of the four weeks. During each week, students took a pre-test on Day 1, and a lecture was then delivered by a certificated simulation lecturer (JC). Students took turns to run face-to-face simulation scenarios on Day 2 led by a certificated simulation lecturer (JC), the students also spent three hours in the skill lab to practice related nursing skills, including but not limited to blood draw, venipuncture, and oral suction on Day 2. Students were then placed in ICU on Day 3 with an assigned RN and patients; a debriefing was conducted at the end of the day. Students were taking other courses on Day 4 and 5.

### Participants

Two cohorts of third-year nursing students (Cohort 2013 and Cohort 2014) taking their four-week training in the ICU from September 2015 through June 2017 were invited to join this study. After acquiring informed consent, one hundred twenty-two students were divided into sixteen groups, with eight groups in each cohort (five to eight students in each group, supplementary Table 1). Students were subsequently assigned to the Integrated and Simulation arms (supplementary Table 1) according to the order in which they entered the ICU internship. Eight groups of students (*n* = 61) were assigned to the Integrated arm, and the other eight groups of students were in the Simulation arm (*n* = 61).

### The procedure

**Fig. 2:**
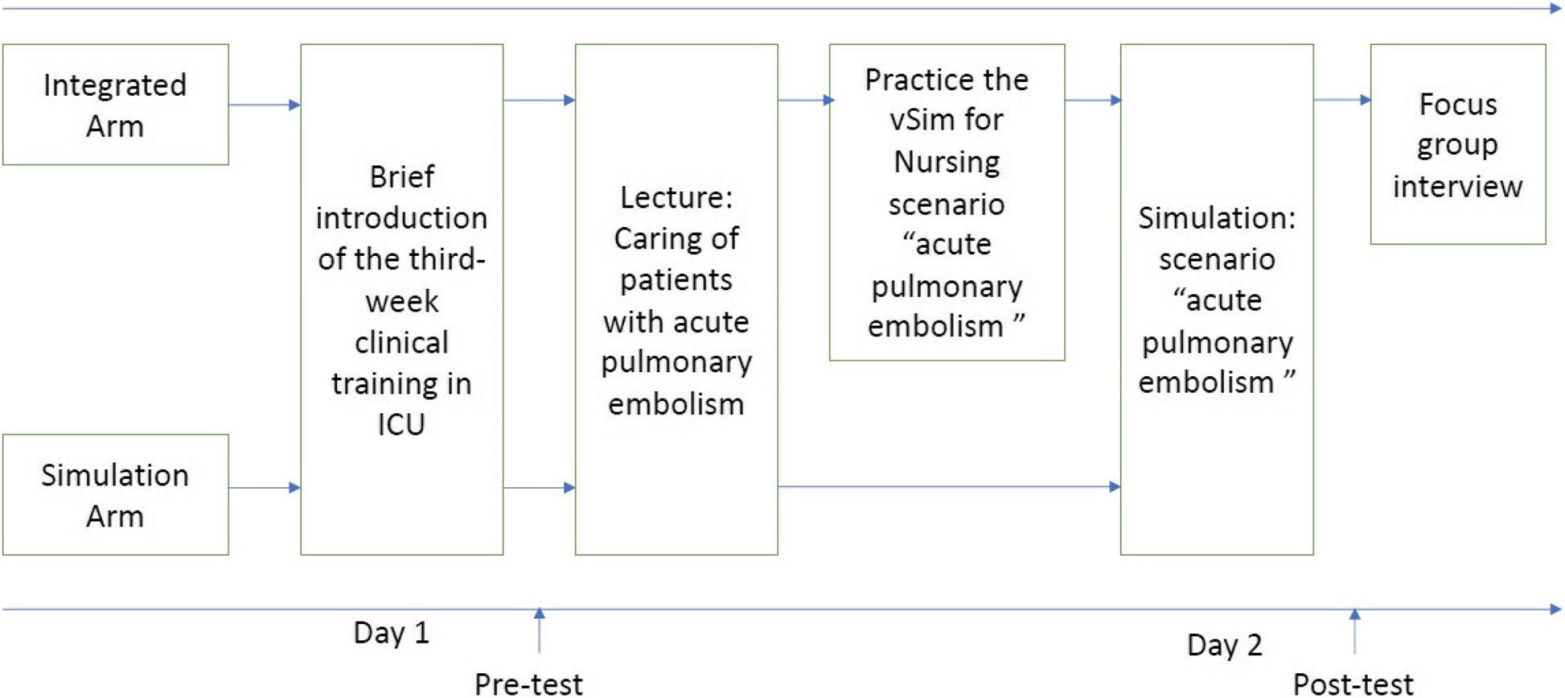
Data collection procedure. Note: Integrated, the non-immersive virtual simulation and high-fidelity face-to-face simulation integrated program arm

Figure 2 illustrates the detailed procedure on the timeline in the quantitative phase. All students completed a pre-knowledge test of acute pulmonary embolism and self-assessment of clinical judgment ability on Day 1 in the third week of the ICU training before the lecture. Only students in the Integrated arm were additionally invited to interact with the vSim for Nursing scenario as a flipped-learning strategy after the pre-test on Day 1. The face-to-face simulation section as a standard component of the ICU training was provided to students in both the Integrated and Simulation arms on Day 2. After the face-to-face simulation on Day 2, a post-knowledge test of acute pulmonary embolism, self-assessment of clinical judgment ability, and evaluation of the simulation were conducted among all students.

#### The vSim

Students in the Integrated arm were provided a user ID and password to access the vSim for Nursing scenario after the pre-test. The scenario, “acute pulmonary embolism,” from vSim for Nursing (Version 1, Wolters Kluwer, Philadelphia, PA, USA) was chosen for the students to complete during the third week of the ICU training. The vSim for Nursing scenario and simulation scenario focus on the initial identification of the signs and symptoms of acute pulmonary embolism post-surgery. The nursing process, including timely assessment, communication with physicians, drug administration, post-assessment, and health education, was also the critical point of this scenario. Students were encouraged to try as many times as they liked and were asked to provide a screenshot to show that they achieved a score of at least 90 out of 100 before the face-to-face simulation on Day 2. Students were told to refrain from discussing the vSim for Nursing nor simulation with each other out of the ICU internship.

#### The face-to-face simulation

Certificated simulation lecturers adopted one scenario (acute pulmonary embolism) from vSim for Nursing (Version 1, Wolters Kluwer, Philadelphia, PA, USA) into a simulation scenario following the standards of best practice for simulation [17]. The lecture team developed the other three face-to-face simulation scenarios following the same standards of best practice, including cases with septic shock, lung cancer, and trauma. Each of the four scenarios was delivered in the four weeks of the ICU training. The simulation scenario was repeated three times, each lasting for one hour, including 15 min of introduction and pre-discussion, 15 min of running the scenario, and 30 min of debriefing after the simulation following our previous protocols [9, 10]. Two or three students (5 to 8 students in each group, supplemental Table 1) took turns to immerse in the simulation scenario.

### Measures

#### Clinical judgment ability

The primary outcome variable, students’ clinical judgment ability, was assessed by the 11-item Lasater Clinical Judgment Rubric (LCJR) [16]. The 11-item LCJR includes effective discovery processes (3 items), valid interpretation of the material (2 items), effective feedback was given (4 items), and effective reflection (2 items). The total score of the LCJR ranges from 11 to 44, with each item rating on a 4-point Likert scale; a higher LCJR score indicates higher self-reported clinical judgment ability. The Cronbach’s alpha of LCJR was 0.953 in the current study.

#### Simulation Design Scale (SDS)

There are 20 items and five domains in this Likert five-point scale, with 5 items in objectives/information, 4 in student support, 5 in problem-solving, 2 in guided reflection and feedback, and 2 in fidelity [20]. The total mean score of SDS ranges from 1 to 5, with each item ranging from 1 to 5 (1 = strongly disagree, 5 = strongly agree). All mean scores greater than 3.5 were considered relatively high for simulation design. The Cronbach’s alpha of the SCS was 0.902 in the current study.

#### Educational practices in simulation scale

There are 16 items and four domains in this Likert five-point scale, with 10 items in active learning, 2 in collaboration, 2 in diverse ways of learning, and 2 in high expectations [21]. The total mean score of this scale ranges from 1 to 5, with each item ranging from 1 to 5 (1 = strongly disagree, 5 = strongly agree). A mean score greater than 4 indicates a high perspective of education practice in simulation. The Cronbach’s alpha was 0.902 in the current study.

#### Student satisfaction and self-confidence in learning

There are 13 items and two domains in this Likert five-point scale, with 5 items in satisfaction and 8 in self-confidence in learning [21]. The total mean score of this scale ranges from 1 to 5, with each item ranging from 1 to 5 (1 = strongly disagree, 5 = strongly agree). A higher mean score indicates a higher level of satisfaction and self-confidence in learning. The Cronbach’s alpha was 0.889 in this study.

### Sample size calculation

The enhanced clinical judgment ability measured by the LCJR was the primary outcome. The sample size for each arm was calculated for a power and a false discovery rate set at 0.8 and 0.05, respectively. The sample size calculations were performed using G*Power 3.1 [22, 23]. For the first hypothesis, we assumed that Simulation has a median effect (Cohen’s *d* = 0.5) [24] on improving clinical judgment ability among nursing students, thus a significant improvement in scores of self-reported clinical judgment ability from pre-test to post-test; thirty-four participants in the Stimulation arm were estimated to test the difference in this assumption. We also hypothesized that the Integrated arm improved more than students in the Simulation arm; fifty-one participants in each arm were estimated to test the difference in this assumption with an effect size of 0.50 (Cohen’s *d*) [24]. In summary, at least 102 subjects should be recruited (51 in each arm) to achieve a power of 0.8 at a significance level of 0.05.

### Data analysis

IBM SPSS Statistics version 25 (IBM Inc., Armonk, NY, USA) was used for data analysis. The frequency, percentage, mean, and standard difference were used for the statistical description of pre-and post-test scores. Pre- and post-test LCJR scores in each arm were compared using a paired t-test to verify the effect of the Integrated program and Simulation on improving students’ clinical judgment ability. The difference between the pre-test LCJR scores, and the post-test LCJR scores from the Integrated and the Simulation arms was compared using an independent t-test to explore if students in the Integrated arm improved more on clinical judgment ability than students in the Simulation arm. The difference between the evaluation for the simulation study from the Integrated and the Simulation arms was compared using an independent t-test to investigate if the students in the two arms perceived differently of the face-to-face stimulation.

### Qualitative phase

The qualitative phase used focus group interviews to collect students' data. The focus group aimed to a) explore novel findings that emerge in the analysis of the quantitative data, b) identify new areas of inquiry in addition to the quantitative assessments, and c) assess the validity of quantitative findings by presenting them for interpretation to study participants in qualitative interviews. Students in the Integrated arm were invited to join the focus group in the qualitative phase after the post-test in a private room. The interview protocol was grounded in the quantitative results from the first phase of the study (Supplementary file 2). To maintain a heightened level of awareness, a journal of personal feeling reflection, and contemplation was kept throughout the study. Eight focus group interviews were conducted, and each interview was audiotaped and transcribed verbatim by a research assistant. Semi-structured interview data analysis followed the process: (a) preparing the data for analysis, (b) conducting content analysis [18], and (c) transforming the textual data into numerical data if possible [25]. This process facilitated the integration of quantitative and qualitative data in a mixed-methods design.

### Integrating the quantitative and qualitative findings

The results of the quantitative and qualitative phases were integrated while comparing the results of the entire study, as well as in the dissection of the study (Fig. 1 for a diagram of the explanatory sequential mixed methods design). Themes extracted from the qualitative phase were compared and contrasted with the subjective performance and clinical judgment measurements in the quantitative phase [18]. Similarity and differences across and with quantitative and qualitative data were presented in tables.

## Results

### Demographic information of the included students

Among the 122 students invited, 105 were females, and 17 were males, aged 19 to 24 (20.65 ± 0.832) years old. All the students invited agreed to participate, on one dropped out. There were no significant differences between the two groups regarding gender and age (*p* = 0.562).

### Quantitative strand findings

### The effect of integrated program and simulation on clinical judgment among nursing students

The scores of clinical judgment ability measured by the LCJR before and after the simulation were presented in Table 1. There was no significant difference regarding the pre-test scores acquired from students in the Integrated and Simulation arms. Both the Integrated and Simulation arms showed significant improvement after the simulation (all *p* < 0.01). Regarding the difference between the Integrated arm and the Simulation arm at post-test, scores in the Integrated arm were significantly higher in five items of two domains (all *p* < 0.05), including effective discovery process and valid interpretation of the material. No significant difference was observed in the other six items of the other two domains (effective feedback was given and effective reflection).

**Table 1:** Comparison of nursing students' scores in each item and domain of the Lasater Clinical Judgment Rubric (LCJR) between pre- and post-test in two arms (*n* = 122)

Items/Domain	Score, Mean (SD)				t^a^	*p*-value
	Integrated (*n* = 61)		Simulation (*n* = 61)			
	Pre-test	Post-test	Pre-test	Post-test		
**1.Focused observations**	1.80 (0.654)	3.25 (0.567) **	1.80 (0.703)	2.79 (0.686) **	4.03	** < 0.001**
**2.Recognizing deviations**	1.98 (0.671)	3.23 (0.462) **	2.10 (0.597)	2.92 (0.331) **	4.28	** < 0.001**
**3.Information seeking**	2.07 (0.442)	3.13 (0.618) **	2.07 (0.680)	2.87 (0.645) **	2.29	**0.024**
**Effective discovery processes**	1.95 (0.421)	3.20 (0.448) **	1.99 (0.399)	2.86 (0.392) **	4.52	** < 0.001**
**4.Prioritizing data**	2.13 (0.427)	3.20 (0.401) **	2.18 (0.592)	2.75 (0.567) **	4.98	** < 0.001**
5.Making sense of data	1.93 (0.602)	3.07 (0.629) **	2.08 (0.737)	2.89 (0.733) **	1.46	0.147
**Valid interpretation of the material**	2.03 (0.427)	3.13 (0.437) **	2.13 (0.507)	2.82 (0.548) **	3.47	**0.001**
6.Calm, confident manner	2.30 (0.843)	3.05 (0.669) **	2.34 (0.981)	3.11 (0.896) **	0.46	0.648
7.Clear communication	2.28 (0.799)	3.21 (0.581) **	2.51 (0.674)	3.02 (0.619) **	1.81	0.073
8.Well-planning intervention	1.92 (0.690)	2.69 (0.593) **	2.18 (0.885)	2.82 (0.785) **	1.04	0.300
9.Being skillful	2.07 (0.834)	2.95 (0.617) **	2.15 (0.872)	3.00 (0.775) **	0.39	0.699
Effective feedback was given	2.14 (0.610)	2.98 (0.465) **	2.30 (0.603)	2.99 (0.562) **	0.13	0.895
10.Evaluation/self-analysis	1.90 (0.625)	2.92 (0.759) **	2.08 (0.714)	2.92 (0.918) **	0.00	1.000
11.Commitment to improvement	1.89 (0.635)	2.95 (0.740) **	2.10 (0.700)	2.77 (1.116) **	1.05	0.295
Effective reflection	1.89 (0.549)	2.93 (0.680) **	2.09 (0.595)	2.84 (0.897) **	0.63	0.533
**Total score**	22.26 (4.644)	33.64 (4.435) **	23.59 (4.383)	31.85 (5.016) **	2.08	**0.039**

Integrated, the non-immersive virtual simulation and high-fidelity face-to-face simulation integrated program arm

^a^comparison of scores in post-test between the Integrated arm and Simulation arm by independent samples t-test

^**^ compared with scores in pre-test by paired samples t-test, *p* < .001

The mean difference of scores between pre and post-test in each item and domain of the LCJR in the two arms were also compared (Table 2). Scores in the Integrated arm significantly improved more than those in the Simulation arm in seven of the 11 items, and three of the four domains of the LCJR, including effective discovery processes (t = 4.82, *p* < 0.05), valid interpretation of the material (t = 5.01, *p* < 0.05), effective reflection (t = 2.10, *p* < 0.05).

**Table 2:** Comparison of the mean difference of participants’ scores in each item and domain of the Lasater Clinical Judgment Rubric (LCJR) between pre and post-test in two arms (*n* = 122)

Items/Domain	Scores, Mean (SD)		t	*p*-value
	Integrated (*n* = 61)	Simulation (*n* = 61)		
**1.Focused observations**	1.44 (0.807)	0.98 (0.764)	3.23	**0.002**
**2.Recognizing deviations**	1.25 (0.722)	0.82 (0.619)	3.50	**0.001**
**3.Information seeking**	1.07 (0.727)	0.80 (0.401)	2.47	**0.015**
**Effective discovery processes**	1.25 (0.512)	0.87 (0.351)	4.82	** < 0.001**
**4.Prioritizing data**	1.07 (0.574)	0.57 (0.670)	4.36	** < 0.001**
**5.Making sense of data**	1.13 (0.645)	0.80 (0.440)	3.28	**0.001**
**Valid interpretation of the material**	1.10 (0.499)	0.69 (0.400)	5.01	** < 0.001**
6.Calm, confident manner	0.75 (0.434)	0.77 (0.761)	0.15	0.884
**7.Clear communication**	0.93 (0.602)	0.51 (0.722)	3.54	**0.001**
8.Well-planning intervention	0.77 (0.529)	0.64 (1.033)	0.88	0.379
9.Being skillful	0.89 (0.551)	0.85 (0.872)	0.25	0.804
Effective feedback was given	0.84 (0.362)	0.69 (0.620)	1.56	0.121
10.Evaluation/self-analysis	1.02 (0.671)	0.84 (0.916)	1.24	0.217
**11.Commitment to improvement**	1.07 (0.727)	0.67 (1.121)	2.30	**0.023**
**Effective reflection**	1.04 (0.601)	0.75 (0.883)	2.10	**0.038**
**Total score**	11.38 (3.661)	8.26 (4.733)	4.07	** < 0.001**

Integrated, the non-immersive virtual simulation and high-fidelity face-to-face simulation integrated program arm

### Nursing students’ feedback on simulation design

Table 3 presents students’ feedback on the simulation in the two arms. All domains in the SDS were rated relatively high both in the Integrated and the Simulation arms, and the difference between these two arms was not significant. However, compared to the Simulation arm, the Integrated arm has higher mean scores in the domains of fidelity (4.09 (SD = 0.588) vs. 3.81 (SD = 0.881), *p* = 0.042).

**Table 3:** Students’ perspective of the simulation (*n* = 122)

Scales/Domains	Score, Mean (SD)		t	*p*-value
	Integrated (*n* = 61)	Simulation (*n* = 61)		
1.Students’ satisfaction and self-confidence (SSS)	4.23 (0.386)	4.18 (0.429)	0.70	0.485
Student satisfaction	4.37 (0.493)	4.24 (0.541)	1.30	0.198
Self-confidence in Learning	4.15 (0.384)	4.14 (0.412)	0.11	0.910
2.Educational practice practices in simulation scale (EPSS)	4.33 (0.401)	4.24 (0.431)	1.16	0.250
Active learning	4.25 (0.435)	4.13 (0.498)	1.45	0.149
Collaboration	4.57 (0.487)	4.63 (0.456)	0.77	0.444
Diverse ways of learning	4.43 (0.442)	4.34 (0.642)	0.90	0.368
High expectations	4.37 (0.605)	4.31 (0.666)	0.50	0.619
3. Simulation Design Scale (SDS)	4.25 (0.387)	4.11 (0.443)	1.74	0.085
Objective/information	4.12 (0.570)	4.00 (0.671)	1.08	0.284
Support	4.23 (0.522)	4.31 (0.364)	1.79	0.077
Problem-solving	4.31 (0.364)	4.25 (0.423)	0.73	0.465
Feedback	4.41 (0.467)	4.30 (0.534)	1.22	0.226
**Fidelity**	4.09 (0.588)	3.81 (0.881)	2.06	**0.042**

Integrated, the non-immersive virtual simulation and high-fidelity face-to-face simulation integrated program arm

Mean scores of educational practice in simulation and each domain were greater than 4 in both arms. Collaboration was the highest, followed by diverse ways of learning, high expectations, and active learning. There was no significant difference between these two arms regarding mean scores of educational practice in simulation and each domain.

Mean scores in satisfaction and self-confidence of students were also greater than 4, with higher scores in the satisfaction domain than in self-confidence in both arms. Regarding scores in satisfaction and self-confidence of students in the two arms, the difference was not significant.

### Qualitative strand findings

The findings of focus group interviews supported the improvement of clinical judgment and helped interpret quantitative data related to these improvements. The vSim for Nursing allowed students to reflect on their decisions in a safe and self-controlled learning environment. Themes and quotes consistent with the domains of the LJCR are listed below and in Table 4. Other advantages, inadequacies, and recommendations were also summarized (Table 4).

**Table 4:** Qualitative findings from focus group interviews in the Integrated arm (*n* = 61)

Findings	Themes	Quotes
**Clinical judgement ability**		
Effective discovery processes	Students can identify critical information they were normally not aware of	*You do not know what to observe at the beginning, but later you know what to discover because the feedback from the vSim for Nursing will lead you to (discover) step by step*
Valid interpretation of the material	Students believed that they could explain and sort out the materials most relevant to patients and evaluate the information when dealing with complex scenarios	*The vSim for Nursing helped me understand what the vital signs mean*
Effective feedback	Students could adjust their actions on time according to the collected information and the feedback from patients	*The vSim for Nursing can give effective feedback quickly. Let you know how to improve, there are many opportunities to improve*
Effective reflection	Students could benefit from the timely reflection provided by the the vSim for Nursing	*Thinking of patients as a whole (person) allows independent analysis of their clinical presentation, reflecting and prudent appraisal of previous experiences, identifying strengths and weaknesses, and continuous improvement*
**Advantages**	The vSim for Nursing improved students' learning interest, leaded students to think actively, and made the boring skill training more vivid	*When I get stuck in my mind (during the vSim for Nursing practice), I will be more impressed if I turn to a book. We can practice by ourselves without the teacher or in the clinic, which is a relatively easy and interesting experience*
**Inadequacies**	There was no oral communication and in-person interaction during the vSim for Nursing practice	*In the vSim for Nursing process, there is no communication with patients, there is no real interaction*
**Recommendation**	Students suggested improving the design and the fidelity and creating more scenarios	*The design of vSim for Nursing itself needs to be closer to the clinical setting* *Only one scenario may make you feel boring. I think more virtual simulation scenarios should be developed*

Integrated, the non-immersive virtual simulation and high-fidelity face-to-face simulation integrated program arm

### Effect on improving clinical judgment

#### Effective discovery processes

Through step-by-step feedback and timely reminders in vSim practice, students can identify the critical learning points and important questions they must know.

*“I think the tone and scene in vSim are authentic; it (vSim) can lead you to believe what to pay attention to when dealing with patients and their diseases. You do not know what to observe initially, but later you know what to discover because the feedback from vSim will lead you to step by step.*

*“vSim allows us to pay attention to the information not covered in the simulation, such as calculating the dosage of drugs and the time interval between drugs. We usually don't pay much attention to the doctor's orders when we do the simulation, so I think combining vSim and simulation is better than just doing a simulation”.*

#### Valid interpretation of the material

Students believed they could explain and sort out the materials most relevant to patients and evaluate and rank the information when dealing with complex scenarios.

*“vSim is a bit strict, but you can know the rules as you need to inquire about allergies before using heparin and the rules of checking, and then we'll apply this to the actual (drug administration) process. However, simulation can also be more flexible. For example, in vSim, when we evaluate pain, we will be with the patients in the private room when we ask questions here and there (stay with the patient). Still, in the actual process, it will be very chaotic (chaotic environment in clinical wards), so the two can be combined with and complementary”.*

#### Effective feedback was given

Students could adjust the treatment regimen on time-based on the information collected by themselves and the feedback from patients and compare them with the knowledge and experience they acquired from previous learning to develop an intervention program.

*“vSim can give effective feedback quickly. Let you know how to improve; there are many opportunities; there is no real tension; quickly master its process.”*

*“…..do vSim you feel like a combination of examination and game, there is a sense of painting, both image memory, but also deepen the body memory”.*

#### Effective reflection

Students could benefit from the timely reflection provided by the vSim.

*“There is no such thing as practicing on real people; we can repeat many times. Suppose there is a violation of nursing principles or some ideas. In that case, there will be reminders on it, as well as sources, that is, we can systematically understand the relevant knowledge and target points of a disease”.*

### Other advantages

#### The subjective initiative of nursing students was improved

The vSim enhanced students' learning interests.

*“Read the book and then do vSim, and you will sometimes find that the knowledge is incomplete. When I get stuck in my mind, I will be more impressed if I turn to a book”.*

#### The teaching form of the course is novel and flexible

The vSim allowed students to choose the time and place freely. The combination of vSim and simulation led students to think actively, stimulated their interest in learning, and made the boring skill training more vivid.

*“I think vSim can save time because we can practice vSim in the dormitory; we do not need to gather in the classroom; sometimes it’s hard to find an available time slot for everyone in the group.”*

*“vSim provides us with such a platform. Without vSim, our main experience might come from clinical and classroom teaching. Now with vSim, we can practice alone without the teacher or in the clinic, which is a relatively easy experience”.*

### Inadequacies and recommendation

Students mentioned that there was no real communication and interaction during vSim practice.

*“In the virtual simulation process, there is no communication with patients; there is no real interaction, can not respond naturally like people. Most of the time, as a reminder, the answer is always a state of emergency.”*

Themes of recommendations to improve the design to combine vSim and simulation also emerged, such as improving the fidelity and creating more scenarios.

*“The design of vSim itself needs to be closer to the clinical setting, to increase the auscultation, palpation and abnormal signs measurement function through computer technology.”*

*“Only one scenario may make you feel bored. I think more vSim scenarios should be developed.”*

### Mixed methods findings

The mixed-methods findings integrated quantitative and qualitative feedback of clinical judgment among nursing students (Table 5). The qualitative quotes provided a context for the quantitative improvement measured by the LJCR. Most of the quantitative findings were confirmed by qualitative findings, including the domains and items in the LJCR. The qualitative quotes expanded quantitative improvement in the domain of “valid interpretation of the material”.

**Table 5:** Integration of clinical judgment improvement from quantitative and qualitative data among nursing students in the Integrated arm (*n* = 61)

Quantitative findings (mean score at pre- and post-test)	Qualitative findings (quote)	Mixed methods interpretation
Effective discovery processes 1.95 (SD = 0.421) vs. 3.20 (SD = 0.448), t = 19.11, *p* < *.001* 1. Focused observations 1.80 (SD = 0.654) vs. 3.25 (SD = 0.567), t = 13.97, *p* < *.001* 2. Recognizing deviations 1.98 (SD = 0.671) vs. 3.23 (SD = 0.462), t = 13.47, *p* < *.001* 3. Information seeking 2.07 (SD = 0.442) vs. 3.13 (SD = 0.618), t = 11.44, *p* < *.001*	Effective discovery processes: The vSim for Nursing + Simulation facilitated the effective discovery processes “When you practice with the vSim for Nursing, for example, if this patient said he had pain, you started monitoring him (vital signs), you assessed his pain, and then you checked him if he has any drug allergies and venous access (IV access), etc. There is a thinking process.” 1. Focused observations: One student mentioned that the disease management process provided by the vSim for Nursing can facilitate the simulation by focusing on observation and collecting useful information from subjective and objective data “When we do it (simulation) virtually, it's like a couple of simulations. It (vSim for Nursing) acts as a guide because it will give you hints about what you need to observe and which information you should focus on.” 2. Recognizing deviations: Students mentioned finding subtle patterns and anomalies in expected patterns and using these results to guide assessments “Everything we did in the simulation (vSim for Nursing) is recorded, (the vSim for Nursing system) told us what needs to be improved, the severity of the error is marked, so you know exactly what the error is, and you'll pay more attention to it.” 3. Information seeking: Students mentioned that the key information can be identified if they missed them before “I think when you do a simulation, sometimes you might miss some details, but the vSim for Nursing has some clues, if you miss something, it (vSim for Nursing) will remind you to find them (information), which I think is pretty good.”	Effective discovery processes: Confirmed 1. Focused observations: Confirmed Students can focus their attention on observation during the vSim for Nursing practice 2. Recognizing deviations: Confirmed Errors were recorded effectively, and the severity of the patients was marked out for students to distinguish and memorize 3. Information seeking: Confirmed The vSim for Nursing can guide students to find the missed information
Valid interpretation of the material 2.03 (SD = 0.427) vs. 3.13 (SD = 0.437), t = 17.21, *p* < *.001* 4. Prioritizing data 2.13 (SD = 0.427) vs. 3.20 (SD = 0.401), t = 14.51, *p* < *.001* 5. Making sense of data 1.93 (SD = 0.602) vs. 3.07 (SD = 0.629), t = 13.70, *p* < *.001*	Valid interpretation of the material: The vSim for Nursing + Simulation helped students avoid fatal errors by improving their understanding of the data “Maybe I will harm them (patients) if I did not practice the vSim for Nursing in advance. The vSim for Nursing could tell you the severity of the error. The vSim for Nursing helped me understand what the vital signs mean; I need to act immediately. Otherwise, the patients will die.” 4. Prioritizing data: The vSim for Nursing provides students with learning opportunities to prioritize problems, feedback on key knowledge points, and how to make decisions, and promotes students to complete data collection “Usually, when I do simulation, I feel that it is not important to ask the patients about their allergy histories before administering medications. However, after practice of the vSim for Nursing, I found that the marks of allergy history are very important. They remind me to pay attention.” 5. Making sense of data: Students were able to further search additional information based on the most valuable data, compare the information with the known rules, and make the right choice “The vSim for Nursing has a correct and pre-set sequence, such as asking about his allergy history and checking his plumbing before we administer medication. This helps us understand the allergy history and vital signs collected.”	**Valid interpretation of the material: Expanded** In addition to accurate interpretation of collected data, the vSim for Nursing also reminded students to pay attention to the crucial findings 4. Prioritizing data: Confirmed By focusing on colleting key issues, the vSim for Nursing helped students prioritize the information collected, addressing the critical changes that affect progress 5. Making sense of data: Confirmed The vSim for Nursing helped students understand what the data mean
Effective feedback was given 2.14 (SD = 0.610) vs. 2.98 (SD = 0.465), t = 18.04, *p* < *.001* 6. Calm, confident manner 2.30 (SD = 0.843) vs. 3.05 (SD = 0.669), t = 13.57, *p* < *.001* 7. Clear communication 2.28(SD = 0.799) vs. 3.21 (SD = 0.581), t = 12.13, *p* < *.001* 8. Well-planning intervention 1.92 (SD = 0.690) vs. 2.69 (SD = 0.593), t = 11.38, *p* < *.001* 9. Being skillful 2.07 (SD = 0.834) vs. 2.95 (SD = 0.617), t = 12.56, *p* < *.001*	Effective feedback was given: Detailed feedback was provided “The feedback from the vSim for Nursing is more detailed than the teacher's feedback in the classroom. For example, when carrying out the doctor's order, the sequence or some clinical considerations are more thoughtful than the simulation.” 6. Calm, confident manner: Students were able to think calmly and calmly when he was in a difficult or complex situation after the vSim for Nursing practice “I think if we do it (simulation) first on the vSim for Nursing, our logistics will be clearer, and then if we do it in real-life simulation, our thinking will be calmer.” 7. Clear communication: Virtual interaction and effective communication with patients, can be supplemented in simulation “The cooperation of medical care (in simulation) is very important. Practice the vSim for Nursing enables us to effectively communicate with the medical team and deal with emergencies and prepare for clinical work.” 8. Well-planning intervention: Students were able to develop well-planned interventions during simulation after they practiced the vSim for Nursing “I think the advantage of practicing the vSim for Nursing before simulation is that you know the whole process and you will be more organized when you do simulation, unlike a chaotic fly with no idea what you should do.” 9. Being skillful: Application of nursing skills during the simulation after practicing the vSim for Nursing can further improve students’ speed and accuracy of nursing skills “The combined learning mode of Virtual simulation and practical simulation can fill the gaps, you know what should be done during the vSim for Nursing practice, i.e., venipuncture and intravenous administration, then you will practice these skills before the simulation and apply them during the simulation. It helped me to be more skilled.”	Effective feedback was given: Confirmed 6. Calm, confident manner: Confirmed Students could constantly practice and became more confident 7. Clear communication: Confirmed Simulation can facilitate effective communication and deal with emergency situations 8. Well-planning intervention: Confirmed The vSim for Nursing + Simulation allowed students to develop effective interventions 9. Being skillful: Confirmed The vSim for Nursing + Simulation facilitated students practicing necessary skills and employing these skills
Effective reflection 1.89 (SD = 0.549) vs. 2.93 (SD = 0.680), t = 13.54, *p* < *.001* 10. Evaluation/self-analysis 1.90 (SD = 0.625) vs. 2.92 (SD = 0.759), t = 11.84, *p* < *.001* 11. Commitment to improvement 1.89 (SD = 0.635) vs. 2.95 (SD = 0.740), t = 11.44, *p* < *.001*	Effective reflection: The vSim for Nursing + Simulation could provide the improvement that should be “It (vSim for Nursing) gives you quick feedback on what you're doing wrong, what you need to do, and so on.” 10. Evaluation/self-analysis: The vSim for Nursing provided timely feedback to students and helped them self-analysis “The vSim for Nursing gave you feedback, from the final evaluation to your practice, you have a chance to improve your learning, and there will be a time to do it (vSim for Nursing) again, everyone will do better so that in turn the vSim for Nursing promotes you to think.” 11. Commitment to improvement: The vSim for Nursing + Simulation help reflect on and evaluate nursing students’ clinical performance. Students could identify key points, consider the best course of action, and demonstrate a desire for improvement “It (vSim for Nursing) can let you know how to do it right in a short time, how to do it is enough, how to be perfect, you can have a lot of opportunities to improve.”	Effective reflection: Confirmed 10. Evaluation/self-analysis: Confirmed The vSim for Nursing + Simulation helped students identify their strengths and weaknesses 11. Commitment to improvement: Confirmed Upon reflectively and judicious evaluation of students’ experience; the vSim for Nursing + Simulation helped students make specific plans to eliminate their weaknesses

Integrated, the non-immersive virtual simulation and high-fidelity face-to-face simulation integrated program arm

## Discussion

The feasibility and effect of a virtual simulation and simulation for improving nursing students’ self-reported clinical judgment ability were established in this mixed-methods study. The findings in the quantitative strand supported all three hypotheses by comparing and verifying the effect of a combination of virtual simulation and face-to-face simulation. The focus group interviews in the qualitative strand yielded different but complementary data, confirming findings in the quantitative strand and providing a context for the quantitative findings.

Improvement after simulation in both arms supported the first hypothesis that both the Integrated program and simulation could enhance nursing students’ self-reported clinical judgment ability (all *p* < 0.01, Table 1), which is in line with previous studies [10, 26–28]. Clinical judgment is the process by which the nurse gathers information, identifies vital information, identifies problems, sets improvement goals, plans and implements interventions, evaluates results, and learns to reflect on them. Participation in simulation requires nursing students to be involved in the process and employ their skills to solve the problems, confirmed by previous studies [9, 10].

Students in the Integrated arm improved more than students in the Simulation arm in scores of effective discovery processes, valid interpretation of the material, and effective reflection (all *p* < 0.05, Table 2), favored the Integrated program and verified our second hypothesis. Table 2 shows the average differences in LCJR scores between pre-and post-test among the two arms, demonstrating that students in the combination of vSim for Nursing and simulation arm reported significantly more improvement in clinical judgment ability than those in the simulation-only arm. These results were consistent with the interview results (see Table 5). Students mentioned in the interview that the combination of vSim for Nursing and simulation could improve communication and operation skills, achieve effective intervention, and improve self-confidence. Evidence showed that students' confidence levels improved with repeated simulation [29]. In addition, previous study shows that students tend to show tension, anxiety, and fear when being assessed and tested in a simulated environment due to the expectation of achieving near-perfect execution [8]. Repeated training in virtual simulation may relieve students’ stress before they immerse in the simulation. However, we did not record the times each student tried the vSim scenario as well as the final score of the vSim scenario, since students in the Integrated arm were provided ample opportunity to get familiar with the vSim scenario, an individual style of learning was also the benefit of the vSim.

Our qualitative findings provided context for the improvement of clinical judgment ability in the Integrated arm. The qualitative data supported the significant improvement in effective discovery processes in the Integrated arm found in the quantitative strand, consisting of focused observations, recognizing deviations, and information seeking (Table 5). Students in the interview session also mentioned that repeatedly conducting vSim for Nursing was like experiencing multiple simulations that enabled them to continuously summarize experiences and rules, supported by a previous study [30]. The vSim for Nursing plays a suggestive role in subsequent simulation, allowing students to collect targeted information, discover outliers, and accurately evaluate the implementation of practical measures, ultimately gradually translating knowledge into clinical thinking and clinical judgment [31].

Mixed-methods findings also supported our hypotheses. The significant improvement of “valid interpretation of the material” and “effective reflection,” especially the prioritizing data, was also mentioned in the interview (Table 5). The sequential evaluation and operation of vSim for Nursing include standardized diagnosis and treatment procedures, and the severity of each error is graded and marked, which enables students to think, distinguish between priorities in the processing process, pay attention to the principle of the problem, and to respond orderly and calmly. In the vSim for Nursing scenario, students interpreted the collected information for further treatment. Thus, virtual simulation helped students think, understand, and apply their previous knowledge [32]. The vSim for Nursing animation is vivid so that students feel different from the classroom teaching [33]. Through the immersive learning of vSim for Nursing, students deepen their multidimensional understanding [34]. Identical to previous findings, this study allowed students to perform virtual exercises repeatedly on-demand before simulation until they were satisfied with their performance [35].

Even though students in the two arms rated different scores in support and fidelity in simulation design, they all rated relatively high when considering their perspective of the simulation, which supported the acceptability of the simulation scenario adopted from the vSim for Nursing, and also supported our third hypothesis. Virtual simulation could be an alternative to improve the fidelity since virtual-reality simulation has the advantage of an entire virtual 3D Platform to present live-action training as closely as possible [31, 36]. Students admitted that the learning objectives were met, they were satisfied with the simulation, and they rated pretty high for simulation design, education practice in simulation, and self-confidence in learning. The vSim for Nursing coupling simulation education for ICU internship bolstered nursing students’ clinical judgment ability by facilitating the translation of knowledge to practice. Maximizing the opportunity for students to participate in clinical judgment that cannot be engaged in clinical practice and to experience clinical case management by engaging in virtual simulation may be an addition to classroom lectures to improve learning outcomes [37].

However, the post-simulation improvement of “effective feedback was given” between the Integrated and the Simulation was not significantly different (Table 2). The main reasons could be that the vSim for Nursing lacks a flexible communication environment, a design to develop students' hands-on ability and real-time observable changes in patients’ conditions. In addition, the vSim was independently practiced by students without a faculty member; the feedback was generated by the system and may have lacked depth. The other reason could be the short training time since the simulation only lasted 1 h.

## Implications

The findings in the current study added a new dimension to nursing education's transition from the face-to-face paradigm to a learner-centered virtual simulation-based education. Although conducted before COVID-19 when in-person learning and face-to-face was the predominant teaching paradigm, this study was particularly important in the context of limited in-person contact during the COVID-19 pandemic and post-pandemic [38]. This study also provided suggestions on how virtual simulation strategies might be improved and what might be developed in future studies through focus interviews. Further studies could also create more virtual reality scenarios since only one scenario was employed in the current study. Only non-immersive virtual simulation was applied in the current; further studies may also develop semi-immersive or immersive virtual simulation and investigate the effect of this virtual simulation on healthcare education. This study only included nursing students taking clinical training in ICU; studies may also explore the effect of the integrated virtual simulation and face-to-face simulation program among nursing students receiving training in other clinical settings.

## Limitations

Several limitations of this study need attention. First, the study period ranged from 2015 to 2017 though we controlled other variables, i.e., using the same teaching material, same instructor, and same settings of the simulation. We used convenience sampling instead of a randomized controlled design, and all students participated in the simulation, which may cover the effect of virtual simulation (vSim for Nursing). In addition, this study was conducted in one center. The clinical judgment ability was self-reported other than acquired by objective assessment, which may impact the findings, and the improvement does not guarantee improvement of patients’ outcomes. The improved clinical judgment ability was measured immediately after the simulation; the improvement may fade after ﻿long periods. We did not compare the two arms in terms of the scores on acute pulmonary embolism and the amount of time spent in the face-to-face simulation. A comparison of the score and the amount of time spent in the simulation may provide more information regarding the effect of the integrated virtual simulation and face-to-face simulation program. Further multicenter randomized controlled studies may recruit a larger sample to validate the current findings. Other studies may also consider adding an objective assessment of clinical judgment ability as well as including patients’ outcomes if possible.

## Conclusions

This mixed-methods study comparatively verified the effect of a combination of virtual simulation and face-to-face simulation for enhancing nursing students’ self-reported clinical judgment ability. Quantitative data analysis supported the clinical judgment improvement, and qualitative data confirmed the quantitative findings. The integrated virtual simulation and face-to-face simulation program provides a forum to practice clinical judgment skills such as critical thinking, communication, and decision-making. Findings from this study may also benefit new graduate nurses in ICU since the scenario was developed for nursing internships in ICU.

### Supplementary Information

**Additional file 1:** **Supplementary file 1.** Good Reporting of A Mixed Methods Study (GRAMMS) checklist. **Supplementary file 2.** Focus group interview protocol.

**Additional file 2:** **Supplementary Table 1.** Distribution of students in two arms (*n*=122).

## Data Availability

Deidentified data will be available upon reasonable request. Requests to access these datasets should be directed to jc22db@fsu.edu.
