# Supplementary material for: Integrated virtual simulation and face-to-face simulation for clinical judgment training among undergraduate nursing students: a mixed-methods study
Source: BMC Med Educ. 2024 Jan 5;24:32. doi: 10.1186/s12909-023-04988-6 (PMC10768231; doi:10.1186/s12909-023-04988-6)
Supplement: Supplementary file 1 — Additional file 1: Supplementary file 1. Good Reporting of A Mixed Methods Study (GRAMMS) checklist. Supplementary file 2. Focus group interview protocol. [file 12909_2023_4988_MOESM1_ESM.docx]

Supplementary File 1

Good Reporting of A Mixed Methods Study (GRAMMS) checklist

| **Guideline** | **Section/page** |
| --- | --- |
| Describe the justification for using a mixed methods approach to the research question | Methods/pg. 7-8 |
| Describe the design in terms of the purpose, priority and sequence of methods | Methods/pg. 7-8 |
| Describe each method in terms of sampling, data collection and analysis | Methods/ pg. 8-13 |
| Describe where integration has occurred, how it has occurred and who has participated in it | Methods/pg. 13-17 |
| Describe any limitation of one method associated with the present of the other method | Discussion/pg. 19-23 |
| Describe any insights gained from mixing or integrating methods | Discussion/pg. 19-23 |

O'Cathain A, Murphy E, Nicholl J. The quality of mixed methods studies in health services research. J Health Serv Res Policy. 2008;13: 92-98.

Supplementary File 2

Focus group interview protocol

Three open-ended questions explored the experience of participating in the integrated program, including “what benefits do you derive from the combination of vSim for Nursing and simulation”, “what advantages do you feel about the combination of vSim for Nursing and simulation”, “what deficits do you feel about the combination of vSim for Nursing and simulation”. One open-ended question depicted students’ suggestions and perceptions regarding improving the design for further training, i.e., “what suggestions or opinions do you have for improving the combination of vSim for Nursing and simulation”. One open-ended question encouraged students to share whatever they would like “do you have any other feelings about this interview”.
