# Supplementary material for: Integrated virtual simulation and face-to-face simulation for clinical judgment training among undergraduate nursing students: a mixed-methods study
Source: BMC Med Educ. 2024 Jan 5;24:32. doi: 10.1186/s12909-023-04988-6 (PMC10768231; doi:10.1186/s12909-023-04988-6)
Supplement: Supplementary file 2 — Additional file 2: Supplementary Table 1. Distribution of students in two arms (n=122). [file 12909_2023_4988_MOESM2_ESM.docx]

Supplementary Table 1

Distribution of students in two arms (n=122)

| Cohort | Semester | Arm | No. of students | Arm | No. of Students |
| --- | --- | --- | --- | --- | --- |
| Cohort 2013 | Fall 2015 | Integrated a | 8 | Simulation A | 8 |
| Cohort 2013 | Fall 2015 | Integrated b | 8 | Simulation B | 8 |
| Cohort 2013 | Spring 2016 | Integrated c | 8 | Simulation C | 8 |
| Cohort 2013 | Spring 2016 | Integrated d | 8 | Simulation D | 7 |
| Cohort 2014 | Fall 2016 | Integrated e | 8 | Simulation E | 8 |
| Cohort 2014 | Fall 2016 | Integrated f | 5 | Simulation F | 8 |
| Cohort 2014 | Spring 2017 | Integrated g | 8 | Simulation G | 7 |
| Cohort 2014 | Spring 2017 | Integrated h | 8 | Simulation H | 7 |

Note:

Integrated, the non-immersive virtual simulation and high-fidelity face-to-face simulation integrated program arm.
